# A Case of Takotsubo Cardiomyopathy Triggered by Asthma Exacerbation After mRNA-based Vaccination for COVID-19

**DOI:** 10.7759/cureus.39499

**Published:** 2023-05-25

**Authors:** Yusuke Tachibana, Tadaaki Yamada, Taisuke Tsuji, Junji Murai, Koichi Takayama

**Affiliations:** 1 Department of Pulmonary Medicine, Graduate School of Medical Science, Kyoto Prefectural University of Medicine, Kyoto, JPN; 2 Department of Respiratory Medicine, Japanese Red Cross Kyoto Daiichi Hospital, Kyoto, JPN

**Keywords:** respiratory failure, mrna-based vaccination, takotsubo cardiomyopathy, asthma exacerbation, covid-19 vaccine

## Abstract

It is recommended to get the multiple coronavirus disease 2019 (COVID-19) vaccinations for almost all people including asthma patients. A 70-year-old Japanese woman with asthma experienced worsening of respiratory symptoms after the second dose of the mRNA-based COVID-19 vaccine BNT162b2. The patient had hypercapnic respiratory failure and cardiac-apex ballooning and was diagnosed with takotsubo cardiomyopathy induced by asthma exacerbation. Therapies for asthma exacerbation resulted in prompt improvement of respiratory failure and cardiac-apex ballooning. Our findings suggest that asthma patients are prone to exacerbations after receiving the COVID-19 vaccination; therefore, stratification of the patients at risk is required.

## Introduction

The coronavirus disease 2019 (COVID-19) pandemic has affected people’s lifestyles, and vaccines have been administered worldwide in order to prevent its infection and aggravation.

Most COVID-19 vaccines are mRNA-based and are highly effective; however, their adverse reactions have not been entirely clarified. However, some studies have reported that COVID-19 vaccination exacerbates asthma [1]. In addition, anaphylaxis, takotsubo cardiomyopathy (TTC), myocarditis, and pericarditis have been reported to occur rarely [2]. Therefore, attention should be paid to the emergence of severe side effects after COVID-19 vaccination.

TTC is an acute, reversible myocardial injury induced by emotional or physical stress. Risk factors for TTC include female sex, menopause, and psychiatric disorders. While natural disasters and negative and positive emotions are mental causes, physical causes include trauma, surgery, medications, or intoxication. Respiratory disorders including pneumothorax, chronic obstructive pulmonary disease exacerbation, and bacterial or viral infections; chemotherapy for cancer; and invasive procedures such as bronchoscopy or intubation have been reported as triggers of TTC in patients with respiratory diseases [3]. Moreover, asthma exacerbation and its therapies, such as short-acting β2 agonists (SABAs), adrenaline, or intubation are known to cause TTC [3-5]. Fatal complications of TTC include cardiogenic shock and cardiac rupture, especially left ventricular wall rupture and ventricular septal perforation; hence, TTC should not be missed. However, because respiratory problems often cause dyspnea or chest pain, TTC is difficult to diagnose [6,7].

Here, we report a case of COVID-19 vaccine-induced asthma exacerbation and TTC in a patient with bronchial asthma. 

## Case presentation

A 70-year-old Japanese woman with a medical history of bronchial asthma, rheumatoid arthritis, and hypertension had been prescribed a fluticasone-vilanterol combination inhaler for asthma and tacrolimus, iguratimod, and golimumab for rheumatoid arthritis. She had not experienced asthma exacerbation during the previous decade. She had never smoked, and had no history of food, drug and any other allergies except pollen allergy. Although she had a slight cough and dyspnea for a few days, she received the second dose of the mRNA-based COVID-19 vaccine BNT162b2 (Pfizer-BioNTech) and took acetaminophen to prevent fever. Her first dose of the COVID-19 vaccine was also BNT162b2, but it did not cause any side effects. Approximately 12 hours after vaccination, her respiratory symptoms worsened, and the patient was transported to our emergency room by ambulance. Her pulse oximetry demonstrated oxygen saturation of 86% (reservoir mask O_2_, 15 L/min). Physical examination demonstrated decreased respiratory sounds in the absence of wheezing. Wheezing emerged after nebulization with the SABA procaterol. The patient had no edema, rash, or aggravation of joint symptoms.

**Figure 1:**
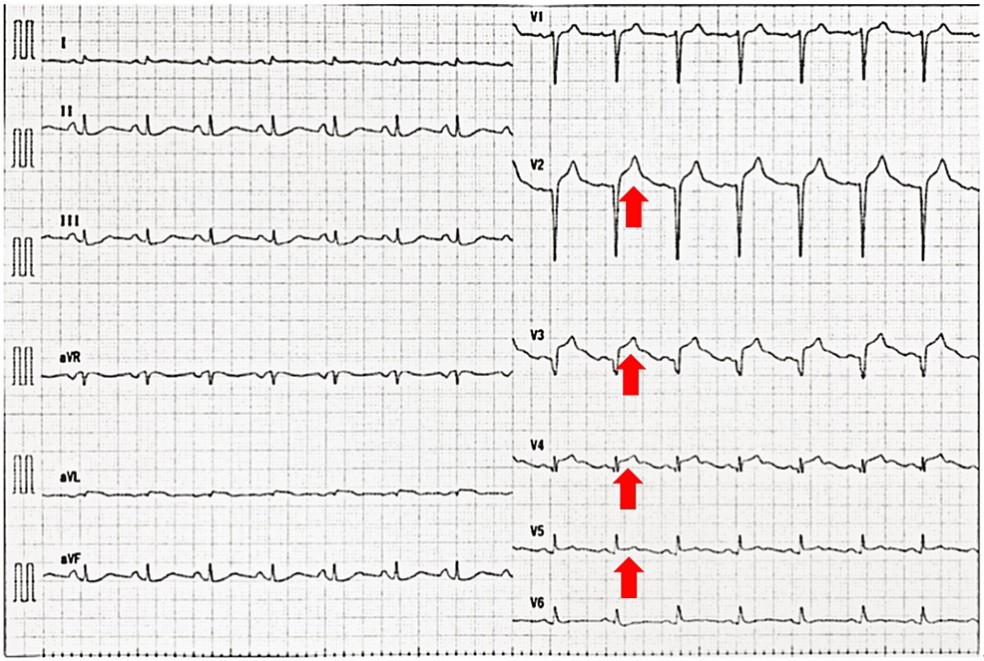
12-lead electrocardiogram The initial 12-lead electrocardiogram of the patient shows ST elevations in V2 - V5 leads.

**Figure 2:**
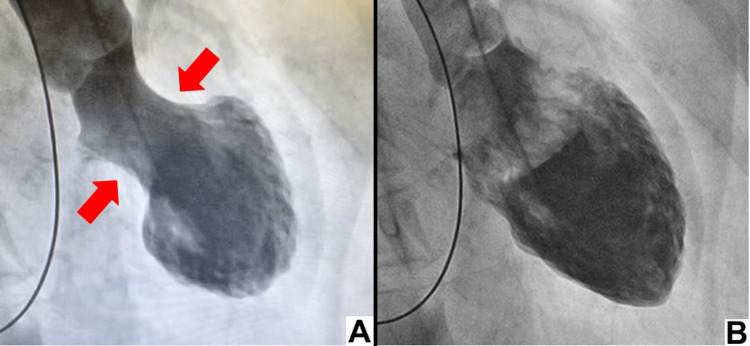
Left ventriculography Left ventriculography of the patient at systole (A) and diastole (B) shows apical akinetic expansion (apical ballooning) and severe hypokinesia of the mid-ventricular segments.

**Figure 3:**
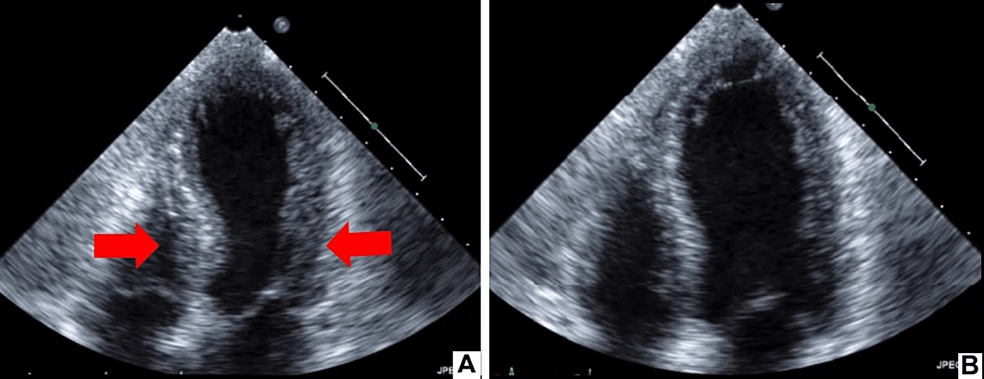
Initial echocardiography Echocardiography of the patient at systole (A) and diastole (B) shows the same motion as the left ventriculography and slightly reduced systolic function (ejection fraction 47%).

The initial blood test showed an increased white blood cell count with eosinophilia, a negative C-reactive protein test, and increased total immunoglobulin (Ig)E. Arterial blood gas analysis before intubation revealed acute hypercapnic respiratory failure (Tables 1-4; reservoir mask O_2_ 15 L/min). A 12-lead electrocardiogram demonstrated sinus rhythm with ST elevation (V2-V5, Figure 1), but the creatine kinase MB and troponin I levels were not elevated. Chest radiography and thoracoabdominal contrast-enhanced computed tomography revealed no acute pulmonary abnormalities except bronchial wall thickening and a calcified nodule in the lower lobe of the right lung. Severe acute respiratory syndrome coronavirus 2 (SARS-CoV-2) nucleic acid test results were negative. In the emergency department, she had a depressed level of consciousness; therefore, endotracheal intubation was performed, and intravenous corticosteroids were administered. To rule out cardiogenic disease, coronary angiography was performed, which revealed no significant coronary artery disease. However, left ventriculography and ultrasonic cardiography revealed apical akinetic expansion (apical ballooning) and severe hypokinesia of the mid-ventricular segments, with slightly reduced systolic function (ejection fraction, 47%, Figures 2-3).

**Table 1:** Initial blood tests (biochemical) before therapy. The total IgE is increased, but creatine kinase MB (CK-MB) and troponin I levels are not elevated. Alb: albumin; ALP: alkaline phosphatase; ALT: alanine aminotransferase; AMY: amylase; AST: aspartate aminotransferase; BUN: blood urea nitrogen; CK-MB: creatine kinase MB; CPK: creatine phosphokinase; Cre: creatinine; CRP: C-reactive protein; Glu: Glucose; IgE: immune globulin E; LDH: lactate dehydrogenase;  T-Bil: total bilirubin; TP: total protein

【Biochemical】	reference range	actual value	unit
LDH	124 - 222	423	U/L
AST	13 - 30	70	U/L
ALT	7 - 23	65	U/L
ALP	106 - 322	114	U/L
TP	6.6 - 8.1	7.4	g/dL
Alb	4.1 - 5.1	4.0	g/dL
T-Bil	0.4 - 1.5	0.6	mg/dL
Glu	73 - 109	297	mg/dL
CPK	41 - 153	176	U/L
AMY	44 - 132	103	U/L
BUN	8 - 20	15	mg/dL
Cre	0.46 - 0.79	0.86	mg/dL
Na	138 - 145	142	mEq/L
K	3.6 - 4.8	4.8	mEq/L
Cl	101 - 108	105	mEq/L
Ca	8.8 - 10.1	9.4	mg/dL
CRP	0 - 0.3	0.19	mg/dL
CK-MB	< 5.0	23	ng/mL
Troponin Ｉ	< 26.2	15.9	pg/mL
Total IgE	< 358	957	IU/mL
specific IgE Japanese cedar pollens	-	Class 4	-
β-D-glucan	< 20	< 6.0	pg/mL
Aspergillus antigen	< 0.5	0.0	-
Cryptococcal antigen	-	negative	-
Candida-mannan antigen	< 0.05	< 0.02	U/mL

**Table 2:** Initial blood tests (blood count) before therapy. No eosinophilia were seen. Bas: basophil; Eos: eosinophil; Hb: hemoglobin; Hct: hematocrit; Lymph: lymphocyte; Mono: monocyte; Neut: neutrophil; PLT: platelet; RBC: red blood cell; WBC: white blood cell

【Blood count】	reference range	actual value	unit
WBC	3,300 – 8,600	12,290	/µL
Neut	39.8-70.5	42.6	%
Lymph	23.1-49.9	45.6	%
Mono	4.3-10.0	4.2	%
Eos	0.6-5.4	6.9	%
Bas	0.3-1.4	0.7	%
RBC	3.86 - 4.92	4.89	×10⁶ /µL
Hb	11.6 - 14.8	15.2	g/dL
Hct	35.1 - 44.4	50.6	%
PLT	15.8 - 34.8	28.8	×10⁴ /µL

**Table 3:** Initial blood tests (coagulation) before therapy. APTT: activated partial thromboplastin time; INR: prothrombin time-international normalized ratio; PT: prothrombin time

【Coagulation】	reference range	actual value	unit
PT	10.3 - 13.0	14.4	sec
INR	0.87 - 1.12	1.3	-
APTT	23.0 - 34.6	30.2	sec
D-dimer	< 1	13.8	μg/mL

**Table 4:** Initial arterial blood gas analysis before therapy. Arterial blood gas analysis (under reservoir mask O_2_ 15 L/min) before intubation shows acute hypercapnic respiratory failure. ABG: arterial blood gas; Lac: lactate; PaCO_2_: partial pressure of arterial carbon dioxide; PaO_2_: partial pressure of arterial oxygen.

【ABG】	reference range	actual value	unit
pH	7.36 - 7.44	6.789	-
PaCO_2_	36 - 44	85.5	mmHg
PaO_2_	75 - 100	206	mmHg
HCO_3_^-^	22 - 26	13.0	mEq/L
Lac	0.56 - 1.39	16	mmol/L

 

Otolaryngological examination revealed that the patient had no eosinophilic sinusitis but had bilateral chronic sinusitis and right secretory otitis media, which were treated with antibiotics. Other infections were excluded, and there were no significant bacterial findings. Serum aspergillus/ cryptococcal/ candida-mannan antigen and β-D-glucan tests were negative. Although the patient had oral candidiasis, it was not a cause of asthma exacerbation.

She had acetaminophen after COVID-19 vaccination; however, she did not have a history of allergy to drugs, including nonsteroidal anti-inflammatory drugs (NSAIDs) and acetaminophen. In addition, the patient did not have any obvious contact with potential allergens. Anaphylaxis was considered in the differential diagnosis, but other cutaneous and mucosal symptoms were absent.

She had not experienced asthma exacerbation during the previous decade. Accordingly, her adherence was not the cause of the asthmatic crisis. In this case, we did not administer adrenaline before the diagnosis of TTC; hence, adrenaline use was not involved in the onset of TTC.

Therefore, we consider that her asthma exacerbation was enhanced by the COVID-19 vaccination and not by allergic reactions or medication.

The patient was treated with procaterol, systemic corticosteroids, leukotriene receptor antagonists, and aminophylline for asthma exacerbation and with heparin for TTC. Adrenaline was used after the diagnosis of TTC. The respiratory status improved promptly, and she was extubated on Day 2 and discharged on Day 10. The improvement of her respiratory status was earlier than her recovery of cardiac function. During hospitalization, ST changes in the electrocardiogram were typical for TTC; ST elevation normalized within a few days and a deep negative T wave developed in a week. After asthma therapy and extubation, the fractional exhaled nitric oxide level was 10 ppb, and spirometry showed an almost normal pattern.

The patient was discharged with instructions to follow up as an outpatient and was prescribed a combination of inhaled corticosteroids/ long-acting β agonists/ long-acting muscarinic antagonists, leukotriene receptor antagonists, and the therapeutic anti-IgE antibody omalizumab. Her outpatient course was stable.

## Discussion

TTC is an acute reversible myocardial injury first reported by Satoh et al. in 1990. The term “takotsubo” means an octopus fishing pot in Japanese that has a round bottom and a narrow neck, and it is similar to the shape of the heart in TTC. TTC is triggered by emotional (negative and positive) or physical stress, including asthma exacerbation. Symptoms similar to those of myocardial infarction have been observed in patients with TTC, without coronary artery stenosis [3,8,9].

The pathophysiology of stress cardiomyopathy is unclear and may involve several mechanisms. One pathway involves high circulating catecholamines released by the sympathetic nerves under stress, including respiratory diseases. Satoh et al. showed that microvascular dysfunction and coronary artery spasms cause TTC [9].

As most patients with TTC are postmenopausal women, estrogen deprivation has been proposed as a cause in several hypotheses, whereas some studies have reported abnormalities in the central autonomic nervous system [10].

Although TTC has a good prognosis, patients with TTC sometimes develop heart failure, arrhythmia, systemic embolism, cardiogenic shock, and cardiac rupture, which might be fatal. Underlying diseases triggering TTC increase mortality (12.2% vs. 1.1% in patients without preexisting diseases) [6]. Our patient did not have cardiac complications; however, adequate treatment for asthma was required.

Dyspnea is a common symptom in respiratory illnesses and TTC; therefore, electrocardiography and echocardiography should be performed before the use of SABAs or adrenaline. 

Infection with SARS-CoV-2 might trigger asthma flare-ups; however, the SARS-CoV-2 polymerase chain reaction test of the patient was negative, indicating that she was not infected with SARS-CoV-2. COVID-19 aggravates many diseases; therefore, vaccines have been developed and implemented rapidly worldwide to reduce the risk of progression and death. mRNA-based COVID-19 vaccines are effective and safe. Nonetheless, adverse events associated with the vaccines are not completely understood. Almost all COVID-19 vaccines used in Japan are mRNA-based.

TTC has been reportedly triggered by mRNA-based COVID-19 vaccines [11-13] or the influenza vaccine [12,14]. However, our patient had asthma exacerbation, and the vaccination did not seem directly of TTC, despite her symptoms being common to both diseases.

Colaneri et al. reported a case of asthma exacerbation triggered by an mRNA-based COVID-19 vaccine [1]. mRNA-based vaccines promote the secretion of type I interferons and increased interferon-I production is associated with asthma exacerbation [15,16]. Nappi et al. reported a case in which two doses of the adenovirus-vectored vaccine ChAdOx1 (Astra Zeneca) progressively worsened asthma and a subsequent dose of the COVID-19 vaccine mRNA-1273 (Moderna) triggered eosinophilic granulomatosis with polyangiitis [17]. Regardless of the type of vaccine, COVID-19 vaccination itself may exacerbate asthma.

Between February 17, 2021, and July 25, 2021, 74,137,348 patients received the BNT162b2 vaccine in Japan. Among these, 219 (0.0003%) cases of asthma attacks were reported to the Japanese Ministry of Health, Labour, and Welfare, which indicates a low incidence [18]. Nevertheless, its molecular biological mechanisms are unclear; therefore, further investigations are warranted to elucidate them.

Hence, we considered that the mRNA-based vaccine might enhance asthma exacerbation and TTC onset in our patient. Our patient was in an immunocompromised state due to rheumatoid arthritis and its treatment with a biopharmaceutical, and infection might have been a trigger of asthmatic crisis; however, it may be less likely given extensive testing. In our case, asthma exacerbation after the vaccination might have been a coincidence. Conversely, it was possible to be an adverse event of the vaccine. However, our study was limited because the relationship between asthma exacerbation and mRNA-based COVID-19 vaccines is currently difficult to demonstrate directly. Thus, more reports and further studies are required.

Our case was previously posted to the Authorea preprint server on January 24, 2023 [19].

## Conclusions

This is a rare case of takotsubo cardiomyopathy triggered by asthma exacerbation after mRNA-based vaccination. It is difficult to demonstrate a relationship between asthma exacerbation and the COVID-19 vaccination, but we cannot deny it. Such a fatal adverse event of the vaccine should be paid attention to, especially in asthma patients.

The vaccination is required to prevent the global pandemic of COVID-19 infection. However, the mRNA-based COVID-19 vaccination can trigger asthma exacerbation and TTC in patients with asthma. This case suggests that some asthma patients are prone to exacerbations after the COVID-19 vaccine; therefore, stratification of the patients at risk, further case reports, and research are required.
